# Searching for serial dependencies in the brain

**DOI:** 10.1371/journal.pbio.3001788

**Published:** 2022-09-07

**Authors:** David Whitney, Mauro Manassi, Yuki Murai

**Affiliations:** 1 Department of Psychology, University of California, Berkeley, California, United States of America; 2 Helen Wills Neuroscience Institute, University of California, Berkeley, California, United States of America; 3 Vision Science Group, University of California, Berkeley, California, United States of America; 4 School of Psychology, University of Aberdeen, King’s College, Aberdeen, United Kingdom; 5 Center for Information and Neural Networks (CiNet), National Institute of Information and Communications Technology, Osaka, Japan

## Abstract

Our senses are barraged with discontinuous and uncertain visual input, but the brain smooths experience by making perception serially dependent. Neural correlates of this have been elusive but this Primer explores a new PLOS Biology study that provides a model for interpreting the complex relationship between physiology and behavior in studies of serial dependence.

## Teaser

Identifying the neural correlates of visual serial dependence has lagged behind the behavioral understanding. A new study in PLOS Biology provides a model of interpreting the complex relationship between physiology and behavior in studies of serial dependence.

**Fig 1:**
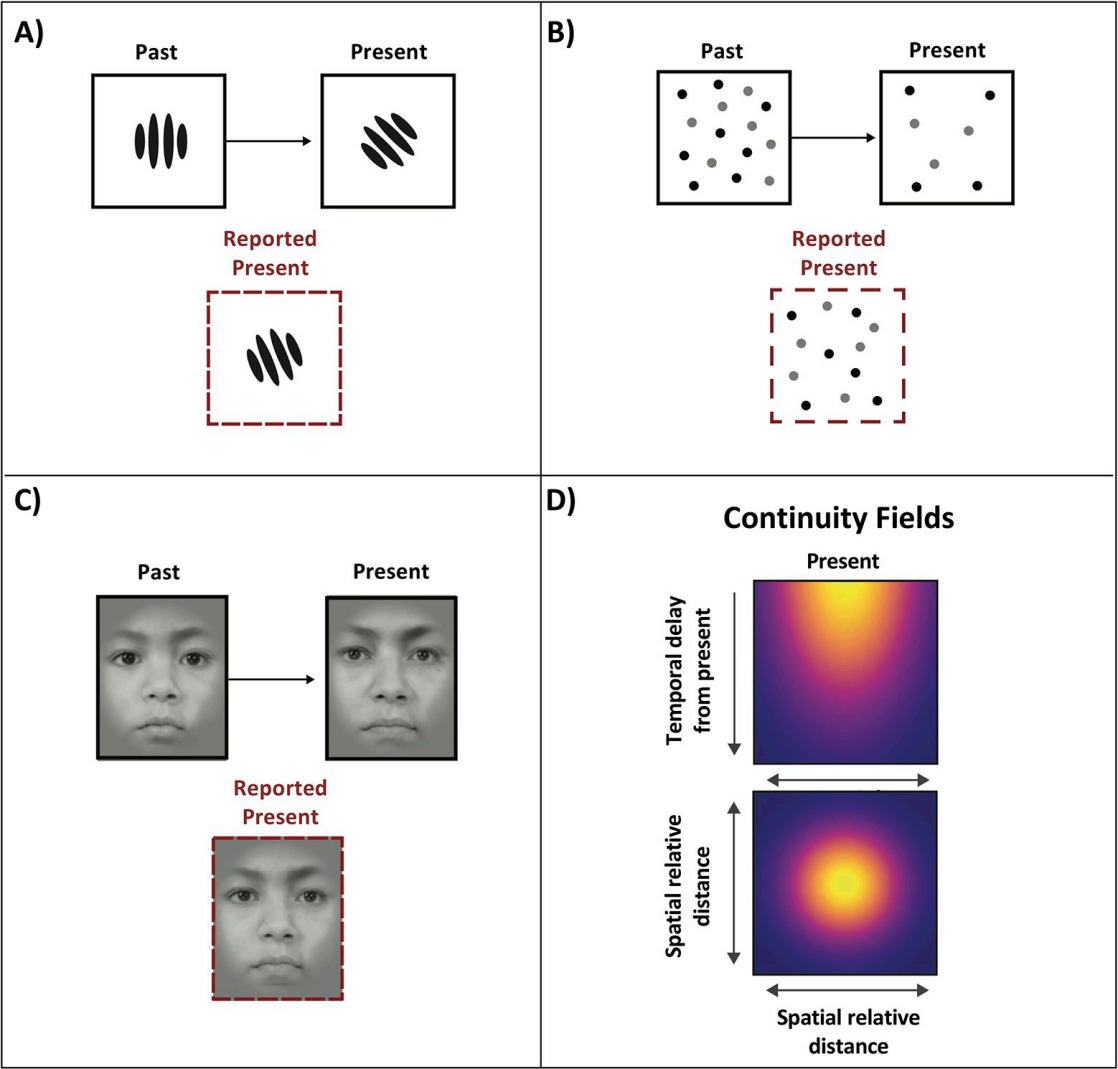
Examples of serial dependency effects, in which perceptions, decisions, and memories are biased, pulled toward the past. (A) Serial dependence in orientation. (B) Serial dependence in numerosity. (C) Serial dependence in face recognition. This is not an exhaustive list; serial dependence occurs for many other stimuli, including at feature and object-selective levels of visual processing, and in other modalities such as audition. (D) Continuity fields: regions of space and time within which the brain treats sequential features and objects as being more similar than they are, for the purpose of temporally smoothing representations. Top panel, temporal tuning. Bottom panel, spatial tuning. Yellow regions show relatively stronger serial dependency. For a demonstration of serial dependence in perception, see https://www.youtube.com/watch?v=cLqVwvdOzuk from [5].

The visual world at our eyes is relentlessly changing around us—images are in constant motion, and there are multiple sources of uncertainty, both external (e.g., occlusions, fog) and internal (e.g., eye blinks, neural noise, processing delays). In principle, our perception should consist of a fluctuating and unstable sequence of visual interpretations, often seemingly unrelated to each other. Yet, our perception of the world and everything in it appears remarkably stable and effortless. This is not accidental: It was recently proposed that the visual system smooths our experience of the world by introducing serial dependence in visual representations [1,2]. This means that images that we encounter over time that are similar to each other will seem even more similar to us than they actually are (Fig 1A–1C). Despite a large array of behavioral papers replicating this serial dependence effect in many domains, the physiological mechanism(s) underlying serial dependence have remained stubbornly unclear. A recent paper in PLOS Biology by Sheehan and Serences (2022) reveals a possible mechanism for serial dependence in the case of orientation perception and provides an important insight on the difficulty of measuring the neural correlates of serial dependence, in general [3].

Serial dependence results when the brain biases information to tie together similar things that occur within a close time period, in order to smooth our experience of the world. Serial dependence affects processing of visual information on many levels, from perception ([4,5]—see Fig 1D) (how do things appear, sound, feel?) to decisions (what color is the coat?, which cup should I grasp?) and memory (did I already look here for my keys?) [6]. In fact, serial dependence probably happens at every level of brain processing, from the earliest [7] to the highest levels [6]. It was recently proposed that the computational goal of serial dependence is to promote more stable interpretations of the world [1]. By recycling prior representations, our brain effectively smooths [4], speeds up, and improves the accuracy of our experience [2].

Because there are so many situations in which serial dependencies occur and have been described behaviorally, no single neural mechanism or module can explain all instantiations of serial dependence. Instead of thinking of serial dependence as a single process or operating at a single level (e.g., only in memory or only in perception), the extensive behavioral work suggests it is a ubiquitous byproduct that emerges at many levels of brain processing. If probed very carefully using behavior and/or physiology at any level, we might therefore find a signature of it. The behavior is relatively clear, and it echoes the computational goal of serial dependence: To smooth our experience of the world, the brain recycles information encountered over the last few seconds [1], within a limited spatial window [1,8], as long as the new information is fairly similar [1,5]. The spatial and temporal range over which the resulting serial dependence happens is called a continuity field [1] (Fig 1D). Continuity fields are spatially and temporally tuned operators within which the brain treats different things as being more similar than they are. The broad range of behavioral findings (e.g., Fig 1) shows that continuity fields may be found at every stage of processing. However, isolating the physiology—the neural implementation—of these behaviorally identified operators has proven notoriously difficult.

Sheehan and Serences (2022) aimed to identify the neural correlates of serial dependence in the orientation domain, which could, in turn, reveal continuity fields for object orientation. This is a good place to start, since much of the modern history of serial dependence began by looking at object orientation. Sheehan and Serences [3] used fMRI to develop a plausible model that links measured neural responses to behavior. This is an important step for linking behavior and physiology in serial dependence research. Their finding was counterintuitive, in that the neural response they measured seems to be opposite the direction of the behavior they were testing. However, they developed a clever model to show how serial dependence can emerge if the brain “reads out” the neural response from the visual cortex and thus predicts a positive association with behavior. The authors have not fully untangled the complexity of what is happening in the brain here, but they did demonstrate an inspired computational architecture about readout that could be extended to many other brain systems and levels of analysis, and it could facilitate other investigations into serial dependence.

The field is still at the stage where the behavioral data outweighs the physiological data. A persistent itch that endures is that there remains no neural signature directly linked to the temporal, spatial, and featural tuning properties of serial dependence despite these well-defined behavioral traits. The paper by Sheehan and Serences (2022) hints at a possible reason for this disparity—the continuity fields may manifest in how information is read out from populations of neurons, or, alternatively, they might be carried by information that is not available from typical amplitude and power-based measurements. What we need are more analytical and neurophysiological tools to understand complex patterns of information processing at the neuron population level in the brain, such as multiplexing, mixed selectivity, oscillatory coding, and others (e.g., [9,10]). Sheehan and Serences (2022) show that using creative computational modeling approaches that can link neural responses to behavior should be central to this goal.

In addition to their clever computational model, Sheehan and Serences (2022) provide an important and optimistic reminder about the value of using behavior as the guide. Behavioral effects—those defined psychophysically—often predate the discovery of the corresponding neural mechanisms. For example, the absorption spectra of photoreceptors, the opponency of color coding in the retina, and the sensitivity of rods to single photons are some of the many processes that were behaviorally demonstrated and defined well before their physiology was understood. In these cases, the neural implementations were identified only when physiological methods caught up, but the computational goal was already there—revealed in the behavioral experiments. We expect a similar discovery pattern for the physiological implementation of serial dependence too.
